# Public and decision-maker stated preferences for pharmaceutical subsidy decisions in Iran: an application of the discrete choice experiment

**DOI:** 10.1186/s40545-021-00365-0

**Published:** 2021-09-06

**Authors:** Gita Afsharmanesh, Farimah Rahimi, Leila Zarei, Farzad Peiravian, Gholamhossein Mehralian

**Affiliations:** 1 Department of Pharmacoeconomics and Pharma Management, School of Pharmacy, Shahid Beheshti University of Medical Sciences, Vali-e-asr, Niayesh Junction, Tehran, Iran; 2 Health Management and Economics Research Center, Isfahan University of Medical Sciences, Isfahan, Iran; 3 Health Policy Research Center, Institute of Health, Shiraz University of Medical Sciences, Shiraz, Iran

**Keywords:** Discrete choice experiment (DCE), Preference, Pharmaceutical subsidy, Developing countries

## Abstract

**Background:**

The argument about funding criteria poses challenges for health decision-makers in all countries. This study aimed to investigate the public and decision-maker preferences for pharmaceutical subsidy decisions in Iran.

**Methods:**

A discrete choice experiment (DCE) was used for eliciting the preferences of the public and decision-makers. Four attributes including health gain after treatment, the severity of the disease, prevalence of the disease, and monthly out of pocket and relevant levels were designed in the form of hypothetical scenarios. The analysis was done by using conditional logit analysis.

**Results:**

The results show all of four attributes are important for pharmaceutical subsidy decisions. But a medicine that improves health gain after treatment is more likely to be a choice in subsidy decisions (by relative importance of 28% for public and 42% for decision-makers). Out of pocket, severity, and prevalence of disease subsequently influence the preferences of the public and decision-makers, respectively. The greatest difference is observed in changing the health gain after treatment and out of pocket levels, between public and decision-makers.

**Conclusion:**

This research reveals that the public is willing and able to provide preferences to inform policymakers for pharmaceutical decision-making; it also sets grounds for further studies.

**Supplementary Information:**

The online version contains supplementary material available at 10.1186/s40545-021-00365-0.

## Background

The scarcity of resources and increasing health care expenditures have made apparent the need for priority-setting and economic evaluation. In the past decade, there has been a growth in health technology assessment as well as in utilizing the economic analysis to shed light on “fourth hurdle” policies at an international level. The decisions related to government and insurer companies have been mandated increasingly to evaluate economically new healthcare interventions [1–3].

In Iran, for the aim of cost-effectiveness, new pharmaceutical products are evaluated formally by Iran Food and Drug Administration (IFDA) assisted by its Economic and Drug Utilization Subcommittees. The IFDA advises the Minister of Health about including medicine in the Pharmaceutical Benefits Scheme [4]. However, there are some rare diseases whose treatments face some hurdles to enter the market and to be put on insurance formularies. To support such patients, provide them with access to effective treatments, and decrease the catastrophic expenditures, the Islamic Republic of Iran's (IRI) government has allocated a budget line named “drug subsidy” to support some serious illnesses. Nonetheless, there is a paucity of solid evidence for prioritizing the allocation of scarce health resources to ameliorate the efficiency and equity of access to pharmaceutical products. On the other hand, the ultimate goal of economic evaluation in the healthcare system is to make decisions about resources that meet the interests of a given society for which they pay and are eligible to benefit from the resources. Furthermore, evidence from studies indicates that nevertheless, cost-effectiveness plays a major role in decision-making but rather, there has been a proliferation of studies suggesting “non-technical” aspects of priority-setting [1, 5, 6]. Due to the public’s increasing interest and engagement in public policies, decision-makers need to be informed of such valuable engagement so that they can make more efficient decisions. Since, in Iran, the public’s preferences, especially the subsidy of pharmaceuticals, are almost thoroughly unknown, this study aims to consider people’s significant views in resource allocation for pharmaceuticals as well as to investigate whether the public and decision-makers preferences for pharmaceuticals subsidization are consistent. Accordingly, the findings of a discrete choice experiment (DCE) as a choice-based technique have been presented in this study.

## Methodology

### Study design

The study employs a DCE model to investigate individual preferences for pharmaceutical resource allocation. DCEs are usually used to recognize people’s preferences in various non-market situations/services/commodities [7–9]; this method has also been broadly employed in health economics [10–15].

### DCEs scenarios

Participants were asked to imagine being a member of a government committee in Iran that is supposed to make decisions regarding pharmaceutical subsidizing. They were told that the pharmaceutical budget was limited, and there were more medicines available that could be funded within the budget; therefore, they had to choose which medicines needed to be funded. Having been provided with the information about two medicines, the respondents were asked to choose the medicine they preferred to get subsidy under the public plan.

Potential attributes which can describe the choice alternatives were identified by a literature review of the debate on decisions for pharmaceutical subsidizing; by so doing, more potential attributes were identified than what could be included in the DCE [15]. The second step incorporated a rigorous analytical approach which was supported by several triangulation and validation practices.

### Study instrument

A DCE was developed by using four different attributes confirmed by literature review and rigorous qualitative study: severity of the disease, health gain after treatment, the prevalence of the disease, and out of pocket for monthly pharmaceutical expenditures. The qualitative study used semi-structured interviews, which were audio-recorded, transcribed, and subject to thematic analysis through 16 key informants' interviews that more details presented in a previous study [16].

Due to the impossibility of presenting respondents with all the possible combinations of choices, a fractional main effect design was chosen, where statistical efficiency of the design was maximized with orthogonality, level balance, and minimal overlap [1, 8]. A summary of the attributes and levels used in the pilot and main DCE is provided in Table 1.

**Table 1:** Final selected attributes and their levels

Attributes	Levels
The severity of disease without treatment	Moderate, severe, without changing in quality of life (QoL)
Health gain after treatment	
	Relative health, full health
Prevalence of the disease	
	Rare, not rare
Cost of treatment per patient for a month	
	Less than 1000,000 Rial (< US$ 30)*; 100,000–2000,000 Rial (US$ 30-60); 2000,000–5000,000 Rial (US$ 60-150); more than 5000,000 Rial (> US$ 150)

*Based on mean currency exchange rate in 2017 according to Central bank of Iran (https://www.cbi.ir/exrates/rates_fa.aspx, access date 04.05.2018)

In this regard, the respondents faced the choice A or B as one sample of a discrete choice experiment question. Table 2 presents an example; for more details see the Additional file 1: Appendix S1.

**Table 2:** An example of a discrete choice experiment question

Medicine	A	B
Attribute		
Severity of disease before treatment	Severe	Moderate
Health gain after treatment	Full health	Without changing the quality of life, only increases the patient's life expectancy, for at least three months
Prevalence of disease	High	Low
Out of pocket	Less than 30 US$	Between 60 and 150 US$
Please select one of the options	A■	B□

After conducting a pilot study using a limited number of questionnaires (*N*=20), in order to confirm the rationality and tradability of options as well as their comprehensibility for people of different ages and levels of knowledge, the main questionnaire was made as follows: the first section covered demographic, economic, and health status information about the respondents. The second part included some general questions about their views on pharmaceutical subsidy in Iran as a warm-up to help the respondents focusing on the subject. Then, 10 scenarios that introduced different attributes and their levels were presented to the participants.

A blank copy of the questionnaire was presented in Additional file 1: Appendix S1.

### Samples and data collection

Then data collection was undertaken in two groups: public and decision-making bodies. The choice sets were included in a self-completion survey. Multistage random cluster sampling was employed to select clusters corresponding to the population in each postal code area. Hence, a total of 500 questionnaires were randomly distributed in the 22 regions of Tehran that these public samples comprised adult members of the general public, whose ages varied from 18 to 75, and whose knowledge was, at least, at the high school level. For the decision-maker sample, the questionnaires were mailed purposively to 65 experts in pharmacoeconomics and pharmaceutical management in Iran who had experience and expertise in resource allocation. Respondents were asked which medicine they preferred to be subsidized, assuming that the health system had enough funds to allocate subsidy to only one of the two medicines and that there were no alternative treatments available. Forced experiments can constrain respondents to express a preference (i.e., make a trade-off among attributes) even when both alternatives are unattractive.

### Data analysis

Choice data were modeled by using a random utility maximization framework [9] with JMP9.0 software. As the data were binary choice data, in each of the choice sets, respondents were asked to choose between two unlabelled alternatives (pharmaceutical A or B), which means conditional logit regressions were used. The goodness of Fit and Wald test were used to check the models.

Assuming that all attributes have an independent influence on respondents’ preference, the following model was estimated:

$$V= {\beta }_{0}+{\beta }_{1 }\mathrm{severity}+{\beta }_{2 }\mathrm{health gain}+{\beta }_{3 }\mathrm{prevalence}+ {\beta }_{4 }\mathrm{out of pocket}+{\varepsilon }_{ij},$$

where $V$ represents the utility associated with the pharmaceutical was specified as a linear function of the attribute levels, ${\beta }_{0}$ represents an alternative specific constant representing the choice of a “new” pharmaceutical (A or B) as opposed to neither and ${\beta }_{1}$ to ${\beta }_{4}$ are the coefficients that indicate the relative importance of each attribute.

## Results

44 questionnaires were collected from policymakers by response rate decision of 67.6%, and for the public respondents, the self-administered questionnaires were continued to 500 completed questionnaires.

### Descriptive statistics

Of the 500 people in the 22 regions of Tehran, 49.2% of the participants were women and their average age was 39.7 ± 14.2. Their first highest degree of education was high school (51%) and the second one was bachelor's degree (34%). The cost pattern is remarkably similar to the pattern of their spending which was reported by the Statistical Center of Iran in 2015. 71.8% of respondents were in a good health state, and 63.8% had a medicine cost of less than US$ 30 per month. Hence, our sample represents a community that was rarely involved in severe illnesses or treatments for either themselves or their families (see Additional file 2: Appendix S2, Table S1).

The result of preliminary questions, suggests that people strongly agreed with allocating subsidies to the most effective medicines (Question 2), and severe illnesses (Question 4). In this regard, there is an agreement between the public and decision-making ideas. The percentage of people who agree with allocating subsidies to expensive medicines (Question 3) is similar in both groups, but for allocating subsidies to rare diseases (Question 8)there was a difference in groups (88% vs. 25%). There is also no consensus on subsidy allocation for medicines that have another effective alternative (Question 5), (95% vs. 62%). The people almost equally agreed with the two questions whose contents were complementary (questions 7 and 10), in other words, one question can be considered as a control question for the other. However, policymakers have shown other results in this regard, (59.1%, 29.5%). The comparison of public and decision-makers’ attitudes is presented in Additional file 2: Appendix S2, Table S2.

### Analysis of respondents’ preferences

The estimated results of the logit model for the two groups are shown in Table 3. The model indicates that for both samples all attributes of the pharmaceutical were significant in explaining the choice variance (*p*≤0.05).

**Table 3:** Logit model estimation for public and decision-makers

Attributes	Levels	Groups	Estimate	Std error	Lower 95%	Upper 95%	L-R Chi Square	DF	Prob > ChiSq
Severity	2–1	Public	− 0.145	0.027	− 0.185	− 0.091	37.829	1	< .0001*
		Decision-makers	− 0.412	0.148	− 0.588	0.077	12.867	1	< .0003*
Health gain	2–1	Public	− 0.783	0.029	− 0.839	− 0.721	1065.588	2	< .0001*
		Decision-makers	− 1.707	0.194	− 1.972	− 1.083	222.960	2	< .0001*
	3–2	Public	0.821	0.044	0.737	0.896			
		Decision-makers	0.863	0.141	0.661	1.089			
Prevalence	2–1	Public	− 0.345	0.024	− 0.395	− 0.297	217.295	1	< .0001*
		Decision-makers	− 0.360	0.104	− 0.633	− 0.086	16.735	1	< .0001*
Cost	2–1	Public	− 0.726	0.048	− 0.818	− 0.617	455.225	3	< .0001*
		Decision-makers	− 0.197	0.156	− 0.655	0.209	40,354	3	< .0001*
	3–2	Public	0.544	0.055	0.440	0.620			
		Decision-makers	− 0.565	0.187	− 0.945	0.400			
	4–3	Public	0.485	0.037	0.409	0.555			
		Decision-makers	0.771	0.190	0.468	1.083			

*means significance level of 0.05

Because the obtained correlation coefficients were less than 0.5, interactions did not enter the model and the orthogonal method were examined the main effects (for more details see Additional file 2: Appendix S2, Table S3). The result of the Total Test of the Model, Goodness of Fit, and Wald test showed the regressor as a whole and the total coefficients in the Logit model were significant and all entered variables were needed (Additional file 1: Appendix S1, Table S4–S6).

**Fig. 1:**
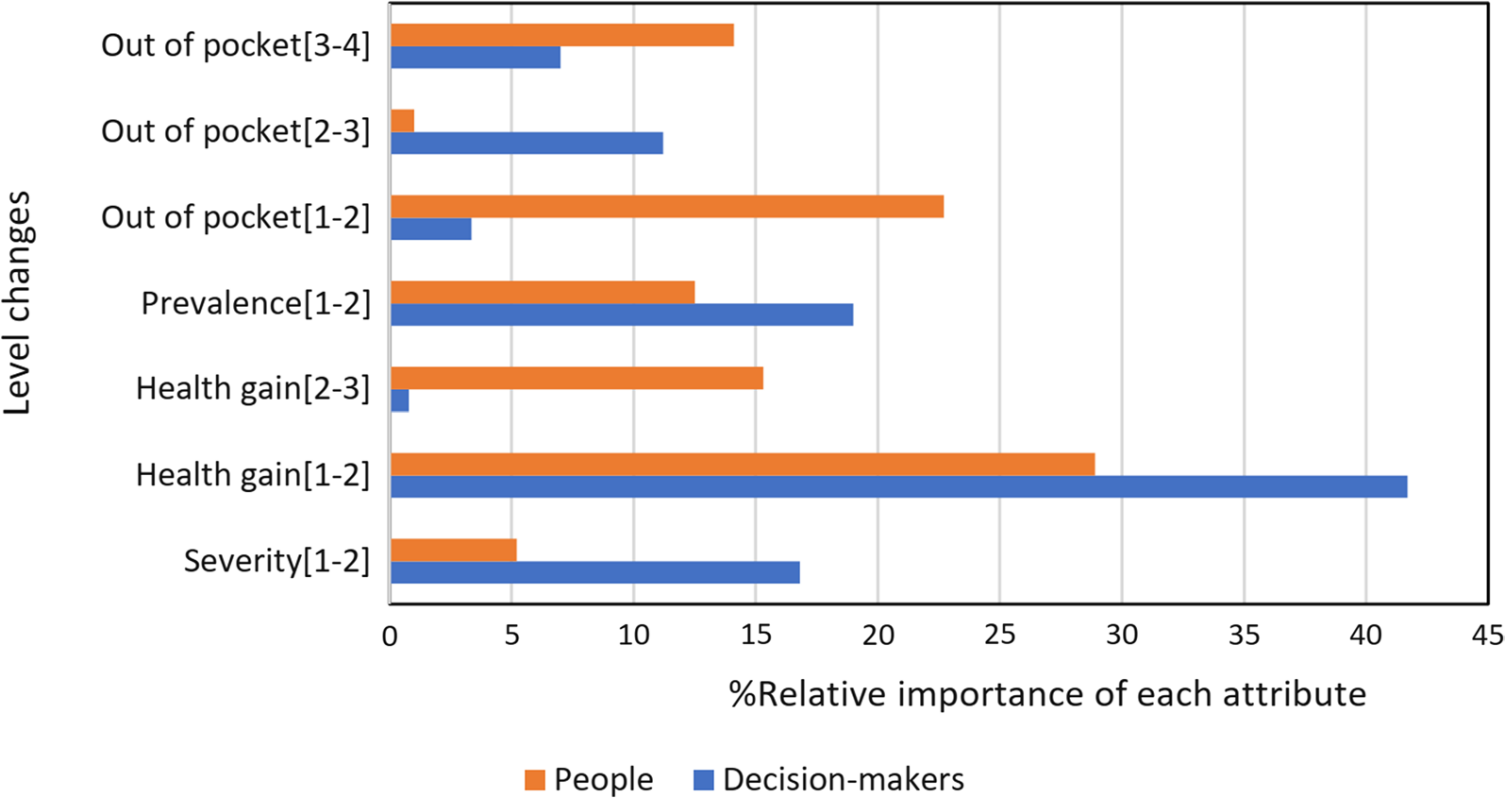
The relative importance of the attributes based on the preferences

In both groups of this study, the highest priority for subsidies ought to be allocated to medicine that can improve the health and quality of life of the patients. Also, the positivity of the signs of “health gain” and “out of pocket” coefficients implies that as these variables increase to higher levels, the preferences of individuals for pharmaceutical subsidy allocation tend to increase. Figure 1 shows the relative importance of each feature in the two sample groups, namely the public and the decision-makers.

With the increasing severity of the disease, the preferences of both groups of public and policymakers to allocate subsidies to the treatment of the disease tend to increase. Therefore, if the other parameters are constant, the change of severity of the disease, from moderate (level 1) to severe (level 2), increased the likelihood that a pharmaceutical would be chosen for funding. About the health gain attribute*,* both public and decision-makers prefer to allocate subsidies to medicines that can enhance people’s health level more significantly, yet it does not mean that they prefer the patients to be fully recovered. In this way, if all other variables remain constant, changing the health gain from 1 to 2 (relative health) and from 2 to 3 (full health), the funding probability in the decision-maker's sample was initially increased and in the next step but it was made constant. However, in the public sample with the same conditions, first, an increase in funding probability was seen, and in the next stage, the utility was decreased.

Disease prevalence in this model indicated that in both groups, increasing the prevalence of the disease increased the likelihood that a pharmaceutical would be chosen for funding.

A noteworthy point regarding the out of pocket for a month attribute was that the first and fourth levels of payment had less likely to be selected for subsidies in both groups, but from the perspective of the public, second and third levels and the decision-makers perspective, the third level of out of pocket for a month increase the likelihood of choosing a pharmaceutical for the subsidy.

While the pattern of results is markedly similar across the two samples, but there are some differences. In the public sample when the disease is severe, not rare, and its relative improvement after treatment is definite; as well as when the out of pocket is from 30 to 60 US$ in the month, maximize the likelihood that a pharmaceutical would be chosen for funding.

In the decision-makers sample, the chance of pharmaceutical subsidy was maximized when the disease is severe, not rare, and its relative or full improvement after treatment is definite; as well as when the out of pocket is from 60-150 US$ in the month.

## Discussion

This is the first study eliciting the stated preferences of the general population for prioritization in health resource allocation in Iran, and ultimately it intends to compare their preferences with those of decision-makers. Furthermore, for the first time, a quantitative trade-off has been undertaken among the most important attributes, with efficiency and equity considerations.

This study was carried out in Iran’s context, and it enjoys a deep exploratory nature; however, remarkable consistency between our findings and those comparable studies undertaken in the UK, Australia, Portugal, and Canada were witnessed [1, 17, 18]. This experiment is useful to introduce a new approach to Iran policy designs.

Also, this study shows the potential of the DCE in identifying a method whereby not only can pharmaceutical suppliers introduce their products onto the market, but also decision-makers can divert resources toward services with the most possible social benefit [1]. Considering the importance of qualitative work on a DCE, we conducted an accurate qualitative study to explore the best attributes and levels to design a responsive DCE model. Nevertheless, to have a more grounded selection of the attributes and their levels as well as the consistency of the results, some more qualitative research could be fruitful [19].

Expecting a low response rate while designing the DCE, we distributed questionnaires individually to increase response rate and to identify response error, and we used the non-random sample as a pilot one. Besides, due to the difficulty of analyzing the alternatives for the public respondents, inevitably, at least high school education was considered as an inclusion criterion, which could be one of the limitations of the design of this study. Respondents from the pilot study showed some unwillingness in answering the DCE choices, which indicates that not only were they engaged in the choice task and its context, but it also highlights their ability to weigh up the difficult choices presented. These offer some confidence in the validity of the experiment [20].

Furthermore, the warm-up test answers were also consensus on the results of DCE. The results show how the public while considering resource allocation for the treatment of others with pharmaceuticals, values the severity of the disease, the amount of health gain from treatment, the prevalence of the disease, as well as the amount of out of pocket for monthly treatment.

Furthermore, the consistency between the pilot and main study models concerning the order of importance of all attributes (as defined by the size of their coefficients in the model) demonstrates the accuracy of findings. Like the findings in other research, whose framework was based on government tax fund payment vehicle, the cost of the pharmaceutical has been proven to be important to the public respondents, while it might be assumed that the cost, might be trivial to make public preferences, especially when the payer is a public resource, rather than the respondents themselves [18]. However, the utility profile of out of pocket for the public and decision-makers are different, which may be due to the budget which decision-makers have in their minds.

Additionally, the findings illuminate that out of pocket is relatively less important than the other attributes, yet it is relevant and there appears to be public willingness to barter out of pocket for effectiveness, no matter whether the out of pocket is publicly supported or not. Unlike others, and in line with Whitty et al. (2008), the government is not expected to buy health gain at any cost [1, 18].

According to this research, the Iranian public view health gain after treatment associated with a new pharmaceutical as the most important consideration when subsidizing pharmaceuticals for the treatment of others suffering a severe or chronic illness. The importance of health gain regarding the treatment of others is consistent with the findings of others exploring British and German preferences about the importance of quality of life (QoL), survival, and chance of success [21–23].

Further, since most respondents have relatively good health, and the cost of their medicines and their families is minimal, the results of the trade-off between out of pocket (OoP) cost and the other attributes may not be biased. Findings show that the concerns of the public should be both-sided, which balances such equity principles as severity and prevalence with efficacy and out of pocket because all attributes were significant [20].

Although this factor can make the findings relevant to policies and present them in a friendly manner to policies, it represents potential limitations in the methodology. Nevertheless, these preliminary findings can be used to provide more detailed information for future study designs [20].

Through more comprehensive research, the indicative and useful results of this study can demonstrate what may be possible in the future.

## Conclusions

This research reveals that the public is willing and able to provide preferences to inform policymakers for pharmaceutical decision-making; it also sets grounds for further studies. The results of this research indicate that the Iranian people appreciate the value of expected health gained from a pharmaceutical drug that is used to treat severe or chronic illnesses. However, in their view, the government is not expected to purchase health gain at any cost. Finally, the findings indicate that compared to other factors such as health gain, the out of pocket is less important to the public, especially when saving people’s life matters, and for whose survival there is virtually no hope. However, it is not insignificant, and it appears that the public is willing to trade cost for effectiveness, even when the payment vehicle is not out of their pocket. All in all, the use of cost-effectiveness as a criterion for decision-making in the process of public funding for pharmaceuticals is supported by this research.

This research can provide the grounds for further studies into public preferences for pharmaceutical funding.

## Supplementary Information

**Additional file 1: Appendix S1.** A blank copy of the questionnaire.

**Additional file 2: Appendix S2.** The additional results.

## Data Availability

All data are available and can be provided by the corresponding author upon rational request.

## Abbreviations

DCE: Discrete choice experiment
IFDA: Iran Food and Drug Administration
IRI: Islamic Republic of Iran's
HTA: Health Technology Assessment
OoP: Out of Pocket
QoL: Quality of Life
QALY: Quality-adjusted life year
