# Supplementary material for: Public and decision-maker stated preferences for pharmaceutical subsidy decisions in Iran: an application of the discrete choice experiment
Source: J Pharm Policy Pract. 2021 Sep 6;14:74. doi: 10.1186/s40545-021-00365-0 (PMC8422609; doi:10.1186/s40545-021-00365-0)
Supplement: Supplementary file 1 — Additional file 1: Appendix S1. A blank copy of the questionnaire. [file 40545_2021_365_MOESM1_ESM.docx]

**Dear respondent,**

By answering the following questionnaire, you are requested to conduct a research study of Ph.D dissertation in the field of pharmacoeconomic and pharmaceutical management at the Faculty of Pharmacy of Shahid Beheshti University of Medical Sciences. As you know, in our country, in addition to the support provided by insurance organizations, an annual budget called drug subsidies is provided by Ministry of Health to compensate for the heavy medical costs of the certain patients. However, due to limited financial resources, the government cannot meet all the existing needs and have been always faced the problem of prioritizing needs. Therefore, during this study, in addition to discovering people's preferences in other surveys, we intend to know if you, as a policymaker, were to subsidize a drug with the limited financial resources at your disposal and make it available to patients free of charge or at a lower cost. Put, how did you decide? And considering the characteristics of each drug or disease in this questionnaire, which one would you prefer and on what basis did you prioritize it? Note that what is important to us is your opinion and there is no right or wrong answer and the information obtained is kept completely confidential. This questionnaire consists of three parts and does not take more than ten or fifteen minutes of your time. Your participation in this project is valuable to us, and you are grateful for giving us your precious time.

Thanks; Gita Afsharmanesh, Ph.D candidate.

**Part 1.** Questions 1 to 10 are mainly about how you feel about the government performance in allocating financial support to the medicines. Please read the questions carefully and choose the option according to your own opinion.

| **No.** | **Attitude** | Exactly agree | Agree | No mention | Disagree | Exactly disagree |
| --- | --- | --- | --- | --- | --- | --- |
| 1 | Drug subsidies should be spent on preventative measures (i.e., preventing large numbers of people in the community or causing more serious complications). |  |  |  |  |  |
| 2 | Drug subsidies should go to the most effective drugs (drugs that further increase the patient's quality of life or longevity). |  |  |  |  |  |
| 3 | Drug subsidies should be given to expensive drugs. |  |  |  |  |  |
| 4 | Drug subsidies should be given to severe illnesses (severe inability to move and perform daily activities) that can be cured. |  |  |  |  |  |
| 5 | Drug subsidies should not be given to drugs that have other effective alternatives. |  |  |  |  |  |
| 6 | Drug subsidies should not be given to foreign drugs that have Iranian equivalents. |  |  |  |  |  |
| 7 | Drug subsidies should only be allocated to lower-income patients. |  |  |  |  |  |
| 8 | Rare diseases (with less frequency) are given priority in the allocation of drug subsidies. |  |  |  |  |  |
| 9 | In allocating drug subsidies, priority is given to chronic diseases that have affected more people in the community. |  |  |  |  |  |
| 10 | Given that drug subsidies are paid from the state budget, they should not be paid exclusively to the poor. |  |  |  |  |  |

**Part 2: Discrete choice experiment questions.**

**Questionnaire guide:**

This section introduces the attributes based on which your preferences are measured, as well as the levels and sizes that are intended for each of them.

Please answer the questions in the next section, which is the main part of this study and contains ten questions, by studying them carefully.

Introducing the features and levels of each of them:

1. Severity of the disease: With this feature, we introduce the quality of the patient's health before treatment or the degree of disability caused by the disease in two levels. In this way, if the person's health is assessed by gait status, pain and discomfort, anxiety and depression, ability to perform normal daily activities (such as work, study, leisure) and personal care (such as bathing), If a person has a severe problem or disability in one of the above conditions (such as hemophilia or some stages of cancer), we consider the person to have a very severe disease, and if he has fewer problems and limitations, but sometimes the disease still prevents life. Becoming normal and interrupting some daily activities is called moderate illness (such as asthma or high blood pressure).

**Note:** In this study, which is about paying subsidies, a low-severity disease (i.e., a condition in which a person's activities are not disrupted despite some pain or other problems) is not considered.

1. The amount of health obtained after treatment: The health obtained as a result of taking the desired drug is predicted in three levels: achieving complete health (ie complete improvement of the patient's problems), relative health (meaning that the patient's problems from The pre-treatment state is reduced but not completely cured (in other words, In other words, severe disease becomes moderate disease and moderate disease becomes very low) and the other state that the drug, without changing the quality of life, only increases the patient's life expectancy, For at least three months. This means that the patient with the same problems and disabilities before treatment, will live longer (at least three months).
2. Prevalence of the disease: means the number of people with the disease in the community. If less than 5 people per 10,000 people are infected (such as thalassemia), we know the prevalence of the disease is low, otherwise we consider the prevalence of the disease to be high.
3. The amount of payment from the patient's pocket: is the amount of money that the patient is forced to pay to receive his medicine, regardless of having or not having insurance, and we pay it in four levels of payment less than 30 US$, between 30 and 60 US$, We have considered between 60 and 150 US$ and more than 150 US$ per month.

To answer the questions in this section, it is assumed that the patient is always a forty-year-old man who suffers from a chronic illness (ie, an illness that lasts more than three months and the duration of treatment is unknown) and to receive a drug subsidy. The drug (drug A or drug B) has been referred to you. And we know that there is no other effective alternative to any of them and the patient only benefits from the basic insurance (i.e. he pays 30% of the price of the insurance medicine) and that with the budget you have, you can only subsidize one of these two drugs. So, you will have to prefer one over the other. In the continuation of this questionnaire, ten binary suggestions are presented which are a combination of different levels of the four features mentioned in the questionnaire guide. Select the one you prioritize for drug to subside. We remind you that there is no right or wrong answer and it is your opinion that matters to us.

**Example**

| **Medicine**  A**ttribute** | **A** | **B** |
| --- | --- | --- |
| **Severity of disease before treatment** | Severe | Moderate |
| **Health gain after taking the medicine** | Relative health | Without changing the quality of life, only increases the patient's life expectancy, for at least three months. |
| **Prevalence of disease** | High | Low |
| **Out of Pocket** | Less than 30 US$, | Between 60 -150 US$ |
| **Please select one of the options.** | A ◼ | B 🞏 |

**Part 3:** Please compare the characteristics described for the two drugs A and B and choose the one you prefer to receive the subsidy.

1.

| **Medicine**  **Attribute** | **A** | **B** |
| --- | --- | --- |
| **Severity of disease before treatment** | Severe | Moderate |
| **Health gain after taking the medicine** | Without changing the quality of life, only increases the patient's life expectancy, for at least three months. | Relative health |
| **Prevalence of disease** | High | Low |
| **Out of Pocket** | More than 150 US$ | Less than 30 US$, |
| **Please select one of the options.** | A 🞏 | B 🞏 |

2.

| **Medicine**  **Attribute** | **A** | **B** |
| --- | --- | --- |
| **Severity of disease before treatment** | Severe | Severe |
| **Health gain after taking the medicine** | Relative health | Without changing the quality of life, only increases the patient's life expectancy, for at least three months. |
| **Prevalence of disease** | High | High |
| **Out of Pocket** | Between 60 - 150 US$ | Between 30 - 60 US$ |
| **Please select one of the options** | A 🞏 | B 🞏 |

3.

| **Medicine**  **Attribute** | **A** | **B** |
| --- | --- | --- |
| **Severity of disease before treatment** | Moderate | Moderate |
| **Health gain after taking the medicine** | Relative health | Without changing the quality of life, only increases the patient's life expectancy, for at least three months. |
| **Prevalence of disease** | High | High |
| **Out of pocket** | Less than 30 US$, | Between 60 - 150 US$ |
| **Please select one of the options** | A 🞏 | B 🞏 |

4.

| **Medicine**  **Attribute** | **A** | **B** |
| --- | --- | --- |
| **Severity of disease before treatment** | Severe | Moderate |
| **Health gain after taking the medicine** | Without changing the quality of life, only increases the patient's life expectancy, for at least three months. | Full health |
| **Prevalence of disease** | Low | High |
| **Out of pocket** | Between 60 - 150 US$ | Between 30-60 US$ |
| **Please select one of the options** | A 🞏 | B 🞏 |

5.

| **Medicine**  **Attribute** | **A** | **B** |
| --- | --- | --- |
| **Severity of disease before treatment** | Moderate | Severe |
| **Health gain after taking the medicine** | Full health | Without changing the quality of life, only increases the patient's life expectancy, for at least three months. |
| **Prevalence of disease** | Low | High |
| **Out of pocket** | Between 60-150 US$ | Less than 30 US$, |
| **Please select one of the options.** | A 🞏 | B 🞏 |

6.

| **Medicine**  **Attribute** | **A** | **B** |
| --- | --- | --- |
| **Severity of disease before treatment** | Severe | Moderate |
| **Health gain after taking the medicine** | Full health | Without changing the quality of life, only increases the patient's life expectancy, For at least three months. |
| **Prevalence of disease** | High | High |
| **Out of pocket** | Between 60 -150 US$ | Less than 30 US$, |
| **Please select one of the options** | A 🞏 | B 🞏 |

7.

| **Medicine**  **Attribute** | **A** | **B** |
| --- | --- | --- |
| **Severity of disease before treatment** | Severe | Moderate |
| **Health gain after taking the medicine** | Full health | Without changing the quality of life, only increases the patient's life expectancy, For at least three months. |
| **Prevalence of disease** | Low | High |
| **Out of pocket** | between 30-60 US$ | Between 60-150 US$ |
| **Please select one of the options** | A 🞏 | B 🞏 |

8.

| **Medicine**  **Attribute** | **A** | **B** |
| --- | --- | --- |
| **Severity of disease before treatment** | Severe | Moderate |
| **Health gain after taking the medicine** | Without changing the quality of life, only increases the patient's life expectancy, For at least three months. | Relative health |
| **Prevalence of disease** | Low | High |
| **Out of pocket** | Between 30-60 US$ | More than 150 US$ |
| **Please select one of the options** | A 🞏 | B 🞏 |

9.

| **Medicine**  **Attribute** | **A** | **B** |
| --- | --- | --- |
| **Severity of disease before treatment** | Severe | Moderate |
| **Health gain after taking the medicine** | Relative health | Full health |
| **Prevalence of disease** | Low | High |
| **Out of pocket** | More than 150 US$ | Between 60-150 US$ |
| **Please select one of the options** | A 🞏 | B 🞏 |

10.

| **Medicine**  **Attribute** | **A** | **B** |
| --- | --- | --- |
| **Severity of disease before treatment** | Moderate | Moderate |
| **Health gain after taking the medicine** | Relative health | Full health |
| **Prevalence of disease** | High | Low |
| **Out of pocket** | Between 30 - 60 US$ | Less than 30 US$, |
| **Please select one of the options** | A 🞏 | B 🞏 |
