# Supplementary material for: Public and decision-maker stated preferences for pharmaceutical subsidy decisions in Iran: an application of the discrete choice experiment
Source: J Pharm Policy Pract. 2021 Sep 6;14:74. doi: 10.1186/s40545-021-00365-0 (PMC8422609; doi:10.1186/s40545-021-00365-0)
Supplement: Supplementary file 2 — Additional file 2: Appendix S2. The additional results. [file 40545_2021_365_MOESM2_ESM.docx]

Table S1: Sample background characteristics compare to general population of Iran.

|  |  | **Number** | **Percent** | **General population of Iran (%) ^a^** |
| --- | --- | --- | --- | --- |
| Total | | 500 | 100 | 100 |
| Gender | Male | 254 | 50.8 | 51.2 |
|  | Female | 246 | 49.2 | 48.8 |
| Age | 18-24 | 68 | 13.6 | 10 |
|  | 25-44 | 257 | 51.4 | 52.6 |
|  | 45-64 | 151 | 30.2 | 32 |
|  | 64-75 | 24 | 4.8 | 5.4 |
| Monthly cost **^b^** | Less than 10milion Rial (US$ 300) | 34 | 6.8 | 7.4 |
|  | 10-20 million Rial (US$ 600) | 174 | 34.8 | 31.4 |
|  | 20-30 million Rial (US$ 900) | 193 | 38.6 | 25.8 |
|  | 30-50 million Rial (US$ 1500) | 70 | 14 | 23.1 |
|  | More than 50 million Rial (US$ 1500) | 15 | 3 | 12.2 |
|  | Missing | 14 | 2.8 | - |
| Education | High school | 256 | 51.2 | - |
|  | Associate in Science (A.S) | 23 | 4.6 | - |
|  | Bachelor of Science (B.S) | 170 | 34 | - |
|  | Master of Science (M.S) | 51 | 10.2 | - |
| Health status | Full health | 359 | 71.8 | - |
|  | Relative health | 115 | 23 | - |
|  | Severe health problem | 22 | 4.4 | - |
|  | Missing | 4 | 0.8 | - |
| Monthly medicine cost | Less than 500000 Rial (US$ 15) | 171 | 34.2 | - |
|  | 500000-1000000 Rial (15-30) | 148 | 29.6 | - |
|  | 1000000-1500000 Rial (30-45) | 49 | 9.8 | - |
|  | 1500000-2000000 Rial (45-60) | 43 | 8.6 | - |
|  | 2000000-5000000 Rial (60- 150) | 47 | 9.4 | - |
|  | More than 5000000 Rial (>150) | 42 | 8.4 | - |
| Family size | 1-2 | 104 | 20.8 | - |
|  | 3-5 | 339 | 67.8 | - |
|  | >5 | 25 | 5 | - |
|  | Missing | 32 | 6.4 | - |

^a^ *According to the Population and Housing Census of Iran in 2016*

*b According to the results of cost and income statistics urban households in 2015*

Table S2: Percentage of agreement with warm-up test in two groups of participants.

| Questions | %Public | %Decision-maker |
| --- | --- | --- |
| Q1. Pharmaceutical subsidies should be used for preventative measures. | 95.8 | 68.2 |
| Q2. Pharmaceutical subsidies should be allocated to the most effective medications. | 91 | 90.9 |
| Q3. Pharmaceutical subsidies should be given to expensive medicines. | 64.3 | 53.5 |
| Q4. Pharmaceutical subsidies should be allocated to severe illnesses. | 83 | 81.8 |
| Q5. Pharmaceutical subsidies should not be given for any medication with another effective alternative. | 62 | 95.5 |
| Q6. Pharmaceutical subsidies should not be granted to any imported medicine having an local manufactured equivalent. | 47.4 | 52.3 |
| Q7. Pharmaceutical subsidies should only be allocated to patients with lower income. | 67.7 | 59.1 |
| Q8. Pharmaceutical products for rare diseases are in priority to receive pharmaceutical subsidies. | 88.3 | 25 |
| Q9. In allocating pharmaceutical subsidies, priority should be given to chronic diseases that afflict more people in the community. | 92 | 63.6 |
| Q10. Due to the fact that pharmaceutical subsidies are paid from the public budget, it should not be granted to the poor exclusively. | 56 | 29.5 |

Table S3: Correlation of Estimates.

| **Corr** | **Severity[1]** | **Health gain[1]** | **Health gain[2]** | **Prevalence [1]** | **OoP[1]** | **OoP [2]** | **OoP [3]** |
| --- | --- | --- | --- | --- | --- | --- | --- |
| **Severity[1]** | 1.0000 | - | - | - | - | - | - |
| **Health gain[1]** | 0.3502 | 1.0000 | - | - | - | - | - |
| **Health gain[2]** | -0.2827 | -0.5213 | 1.0000 | - | - | - | - |
| **Prevalence[1]** | 0.1367 | 0.2183 | -0.1956 | 1.0000 | - | - | - |
| **OoP [1]** | 0.0550 | 0.1529 | -0.0203 | -0.0267 | 1.0000 | - | - |
| **OoP [2]** | -0.0739 | 0.0866 | -0.0457 | -0.0783 | -0.2213 | 1.0000 | - |
| **OoP [3]** | -0.3763 | -0.1779 | 0.2999 | -0.0343 | -0.2241 | -0.1616 | 1.0000 |

Table S4: Whole Model Test.

| **Model** | **-Log Likelihood Public** | **-Log Likelihood Decision-makers** | **DF** | **Chi-square**  **Public** | **Chi-square**  **Decision-makers** | **Prob>ChiSq** |
| --- | --- | --- | --- | --- | --- | --- |
| Difference | 1174.8468 | 195.69231 | 7 | 2349.694 | 391.3846 | <.0001* |
| Full | 5728.8989 | 401.79824 |  |  |  |  |
| Reduced | 6903.7457 | 597.49055 |  |  |  |  |

*For “Public”: RSquare (U)= 0.1702; AIC_C_= 11473.8; BIC= 11531.4; Observations (or Sum Wgts)=9960. For “Decision-makers”: RSquare (U)=* 0.3275*; AIC_C_=* 819.765*; BIC=* 857.671*; Observations (or Sum Wgts)=* 862*.*

Table S5. Lack of Fit Test.

| **Source** | **DF** | **-log Likelihood Public** | **-log Likelihood Decision-makers** | **Chi-square Public** | **Chi-square Decision-makers** |
| --- | --- | --- | --- | --- | --- |
| **Lack Of Fit** | 11 | 464.4416 | 53.63234 | 928.8833 | 107.2647 |
| **Saturated** | 18 | 5264.4573 | 348.16590 | Prob>ChiSq |  |
| **Fitted** | 7 | 5728.8989 | 401.79824 | <.0001* |  |

Table S6. Effect Wald Test.

| **Source** | **Nparm** | **DF** | **Wald ChiSquare Public** | **Wald ChiSquare Decision-makers** | **Prob>ChiSq Public** | **Prob>ChiSq Decision-makers** |
| --- | --- | --- | --- | --- | --- | --- |
| **Severity** | 1 | 1 | 6.12913944 | 6.7563784 | 0.0133* | 0.0093* |
| **Health gain** | 2 | 2 | 1307.41423 | 205.76572 | <.0001* | <.0001* |
| **Prevalence** | 1 | 1 | 296.880214 | 18.4780766 | <.0001* | <.0001* |
| **Cost** | 3 | 3 | 462.003204 | 28.9547268 | <.0001* | <.0001* |
